# Supplementary material for: Patterns of Intron Gain and Loss in Fungi
Source: PLoS Biol. 2004 Nov 30;2(12):e422. doi: 10.1371/journal.pbio.0020422 (PMC532390; doi:10.1371/journal.pbio.0020422)
Supplement: Table S1 — Also available at http://genes.mit.edu/NielsenEtAl/. (4.3 MB ZIP). [file pbio.0020422.st001.zip › NielsenEtAl/html/646.html]

AN8853.1.NCU00410.1.MG06266.1.FG06927.1


```
 CLUSTAL W (1.82) Multiple Sequence Alignments - Introns Inserted


Sequence 1: MG06266.1	436 aa
Sequence 2: FG06927.1	440 aa
Sequence 3: NCU00410.1	434 aa
Sequence 4: AN8853.1	445 aa
Alignment Length: 446 aa
Number Identitical Residues: 351 aa
Alignment Score (without introns) 14116


MG06266.1 	-MSEPQGNEAEKN0IEIWKVKKLIKRLEAARGNGTSMISLIIP1PKAQISQAAKMLAEEY
NCU00410.1	--MDPNANEAEKN0IEIWKVKKLIKRLEAARGNGTSMISLIIP1PKDQISRAAKMLAEEY
FG06927.1 	-MSDAQANEAEKN0IEIWKVKKLIKRLEAARGNGTSMISLIIP1PKDQVSRAAKMLAEEY
AN8853.1  	MSTVQQANEAEKN~IEIWKIKKLIKRLEAARGNGTSMISLIIP1PKDQVSRAAKMLAEEF
          	     :.****** *****:*********************** ** *:*:********:

MG06266.1 	0GTASNIKSRVNRQSVLSAITSTQQRLKLYNKVPPNGLVVYCGEILTSEGKERKVNIDFE
NCU00410.1	0GTASNIKSRVNRQSVLSAITSTQQRLKLYNKVPPNGLVVYCGEILTSEGKERKVNIDFE
FG06927.1 	0GTASNIKSRVNRQSVLSAITSTQQRLKLYNKVPPNGLVVYCGEILTQEGKERKVNIDFE
AN8853.1  	0GTASNIKSRVNRLSVLSAITSTQQRLKLYNKVPPNGLVVYCGEIITSEGKERKINIDFE
          	 ************ *******************************:*.******:*****

MG06266.1 	PFKPINTSLYLCDNKFHTEALAELLESDQKFGFIIMDGNGALFGTLSGNTRDIVHKFSVD
NCU00410.1	PFKPINTSLYLCDNKFHTEALAELLDSDQKFGFIIMDGNGALFGTLSGNTRDIVHKFSVD
FG06927.1 	PFKPINTSLYLCDNKFHTEALAELLESDQKFGFIIMDGNGALFGTLSGNTREVVHKFSVD
AN8853.1  	PFKPINTSLYLCDNKFHTEALSELLESDQKFGFIIMDGNGALFGTLSGNTREILQKLSVD
          	*********************:***:*************************::::*:***

MG06266.1 	LPKKHGRGGQSALRFARLREEKRHNYVRKVAELAVQNFITADKVNVAGLILAGSADFKND
NCU00410.1	LPKKHGRGGQSALRFARLREEKRHNYVRKVAELAVQCFITNDRPNVAGLVLAGSADFKND
FG06927.1 	LPKKHGRGGQSALRFARLREEKRHNYVRKVAELAVQNFITADKVNVAGLILAGSADFKSD
AN8853.1  	LPKKHGRGGQSALRFARLREEKRHNYVRKIAELAVQNFITNDKVNVAGIILAGSADFKND
          	*****************************:****** *** *: ****::********.*

MG06266.1 	LNASDMFDGRLATKVIKVVDVSYGGENGFNQAIELSSETLGNVKFIQEKKLIGKYFEEIS
NCU00410.1	LNASDLFDPRLQTKVVKVVDVSYGGENGFNQAIELSSETLGNVKFIQEKKLIGKYFEEIS
FG06927.1 	LNASDLFDNRLATKVIKVVDVSYGGENGFNQAIELSSETLGNVKFIQEKKLIGKYFEEIS
AN8853.1  	LNQSDMFDQRLQAKVIKVVDVSYGGENGFNQAIELSAETLSNVKFVQEKKLIGKYFEEIS
          	** **:** ** :**:********************:***.****:**************

MG06266.1 	QDTGRICYGVEDTLKALELGAVETLIVFENLEITRWTLKDSTGTEIILHTTKEQETGNRD
NCU00410.1	QDTGRVCYGIEDTLKALELGAVETLIVFENLEVNRWVLKDSNGSEVILHTTKAQEQASRD
FG06927.1 	QDTGKVCYSIEDTLKALELGAVETLIVFENLEITRWVLKDNNGSEIILHTTKQQEQSNRD
AN8853.1  	QDTGKVCYGVEDTLKALELGACETLIVYENLDVTRWVLKNSEGNEVVVHTTKAQEE-NKE
          	****::**.:*********** *****:***::.**.**:. *.*:::**** **  .::

MG06266.1 	QFMDKETGQEMEVVNQESFLEWVAEHYKDFGTALEFVSDRSTEGNQFVKGFGGIGGLLRY
NCU00410.1	AFMDKETGQEMEVVSQESFLEWIAEHYKDFGATLEFVSDRSTEGNQFVKGFGGIGGILRY
FG06927.1 	KFLDKETGQEMEIVSQESFLEWIAEHYKDFGTTLEFVSDRSTEGNQFVKGFGGIGGLLRY
AN8853.1  	FFLDKDTGAEMEVVDQSSFLEWLAENYKDFGATLEFVSDKSSEGNQFVKGFGGIGAILRY
          	 *:**:** ***:*.*.*****:**:*****::******:*:*************.:***

MG06266.1 	KVNFEQLAEVDDDDDYYDGM---------
NCU00410.1	KVNFEQLADVSDDDEYYDD----------
FG06927.1 	KVNFEQLADVSDDDEYYDGELTTL-----
AN8853.1  	KVNFEQLADYSDEDEFYDGRCTMLLSILP
          	********: .*:*::**.  :   :  .
```
